# Influence of Brewer’s Spent Grain Compounds on Glucose Metabolism Enzymes

**DOI:** 10.3390/nu13082696

**Published:** 2021-08-04

**Authors:** Daniela Becker, Tamara Bakuradze, Marcel Hensel, Simone Beller, Carolina Corral Yélamos, Elke Richling

**Affiliations:** Department of Chemistry, Division of Food Chemistry and Toxicology, Technische Universität Kaiserslautern, Erwin-Schrödinger-Straße 52, 67663 Kaiserslautern, Germany; bakuradze@chemie.uni-kl.de (T.B.); mhensel@rhrk.uni-kl.de (M.H.); sbeller@rhrk.uni-kl.de (S.B.); carolinacorralyelamos@gmail.com (C.C.Y.)

**Keywords:** brewer’s spent grain, glucose metabolism, bioactives, polyphenols

## Abstract

With a yearly production of about 39 million tons, brewer’s spent grain (BSG) is the most abundant brewing industry byproduct. Because it is rich in fiber and protein, it is commonly used as cattle feed but could also be used within the human diet. Additionally, it contains many bioactive substances such as hydroxycinnamic acids that are known to be antioxidants and potent inhibitors of enzymes of glucose metabolism. Therefore, our study aim was to prepare different extracts—A1-A7 (solid-liquid extraction with 60% acetone); HE1-HE6 (alkaline hydrolysis followed by ethyl acetate extraction) and HA1-HA3 (60% acetone extraction of alkaline residue)—from various BSGs which were characterized for their total phenolic (TPC) and total flavonoid (TFC) contents, before conducting in vitro studies on their effects on the glucose metabolism enzymes α-amylase, α-glucosidase, dipeptidyl peptidase IV (DPP IV), and glycogen phosphorylase α (GPα). Depending on the extraction procedures, TPCs ranged from 20–350 µg gallic acid equivalents/mg extract and TFCs were as high as 94 µg catechin equivalents/mg extract. Strong inhibition of glucose metabolism enzymes was also observed: the IC_50_ values for α-glucosidase inhibition ranged from 67.4 ± 8.1 µg/mL to 268.1 ± 29.4 µg/mL, for DPP IV inhibition they ranged from 290.6 ± 97.4 to 778.4 ± 95.5 µg/mL and for GPα enzyme inhibition from 12.6 ± 1.1 to 261 ± 6 µg/mL. However, the extracts did not strongly inhibit α-amylase. In general, the A extracts from solid-liquid extraction with 60% acetone showed stronger inhibitory potential towards a-glucosidase and GPα than other extracts whereby no correlation with TPC or TFC were observed. Additionally, DPP IV was mainly inhibited by HE extracts but the effect was not of biological relevance. Our results show that BSG is a potent source of α-glucosidase and GPα inhibitors, but further research is needed to identify these bioactive compounds within BSG extracts focusing on extracts from solid-liquid extraction with 60% acetone.

## 1. Introduction

In recent decades, there has been growing interest in the valorization of agri-food waste and agricultural by-products as a way of achieving sustainable food production. At present, such by-products are mainly used as fuels, organic fertilizers, or animal feed. However, they could also be valuable sources of bioactive compounds. Consequently, there is ongoing research on their potential uses in the pharmaceutical industry and in functional foods [1]. One byproduct available in very large quantities is brewer’s spent grain (BSG), the solid fraction of barley malt remaining after wort production; up to 39 million tons of BSG are produced annually, with 3.4 million tons being generated within the European Union (EU) [2,3]. It is reported to be rich in protein (19–30% *w*/*w*) and fiber (30–50% *w*/*w*) and is therefore currently used as a low-cost cattle feed, but it could also be used to improve the nutritional value of human food products. Recently, Pratap Singh et al. tested different drying methods of BSG to find a sustainable and sensory appealing method with the aim of using BSG as protein rich snack [4]. Sahin et al. investigated two protein or fiber rich products prepared from BSG in enhancing the nutritional value of pasta or even improving the pasta quality [5]. Further studies also have investigated its use as an additive to increase the mineral, protein, and fiber content of baked foods [6,7]. BSG also contains relatively large quantities of lipids and polyphenols, mainly hydroxycinnamic acids, but also lignans, hydroxybenzoic acids, and flavonoids such as catechins [8,9,10,11]. It is particularly rich in hydroxycinnamic acids, which form part of the cell wall structure and can be released in concentrations of about 220 mg/100 g BSG by alkaline treatment [8]. The antioxidant activity of such polyphenols has been studied extensively in vitro and in vivo, and their effects have been attributed to factors including their radical scavenging ability, modulation of enzymatic activity, and ability to affect signal transduction pathways [12,13,14]. In addition, hydroxycinnamic acids have been found to inhibit glucose metabolism enzymes such as α-amylase and α-glucosidase [15,16,17], making them an attractive research topic due to increasing incidence and prevalence of diabetes type 2 [18]. For instance, some cinnamic acid derivatives were shown to be potent inhibitors of α-glucosidase from yeast and rat in vitro [15,16,19], and of pancreatic porcine α-amylase [17]. These enzymes play important roles in digestion of nutritional polysaccharides; their inhibition reduces glucose liberation and thus lowers blood glucose levels. α-Amylase is found in the saliva and the duodenum, and catalyzes the hydrolysis of α-1,4-glucan bonds in starch, maltodextrins, and malto-oligosaccharides. This is followed by a hydrolytic reaction that liberates α-glucose from the non-reducing end of α-glucose residues, which is catalyzed by α-glucosidase in the small intestine. Both enzymes are already pharmaceutical targets in the treatment of diabetes type 2, as exemplified by the anti-diabetic agent Acarbose [19]. Additionally, a strong in vivo antidiabetic effect was observed in a 30-day feeding study in which type 2 diabetic rats were given 50 mg ferulic acid/kg body weight. Various mechanisms were hypothesized to explain this outcome, including enhancement of insulin signaling and inhibition of gluconeogenesis [20]. Strong antidiabetic effects were also observed in another 30-day feeding study with 50 mg ferulic acid/kg body weight in type 2 diabetic rats, and it was shown that the treatment affected the activity of various glycogen metabolism enzymes including glycogen synthase (GS), glucokinase (GK), and glycogen phosphorylase (GP). Diabetic animals exhibit reduced activity of glycogenesis enzymes (GS and GK) and elevated activity of those involved in glycogenolysis (GP); these activity levels were normalized by ferulic acid to a degree similar to that induced by treatment with the antidiabetic drug metformin. Inhibition of GP in the liver is a potent target for the management of type 2 diabetes [21]. Whole grain was also found to influence glucose metabolism in humans: a wholegrain cereal-based diet reduced postprandial insulin and triglyceride levels in men and women with metabolic syndrome [22]. Furthermore, protein hydrolysates made from BSG using different enzyme preparations, were proven to be potent inhibitors of α-glucosidase and dipeptidyl peptidase IV (DPP IV) in vitro. DPP IV plays an important role in insulin secretion because it catalyzes the degradation of the incretin hormone glucagon-like peptide-1 (GLP-1), which stimulates post nutrient insulin secretion and thus facilitates glucose uptake into cells. DPP IV inhibitors such as Sitagliptin, which is an active ingredient in diabetes drugs, thus reduce GLP-1 degradation and thereby indirectly enhance cellular glucose uptake [23]. We therefore investigated the effect of BSG extracts on human glucose metabolism. Extracts prepared by conventional solid-liquid extraction with 60% acetone were compared to extracts prepared by alkaline hydrolysis, which reportedly releases bound phenolic acids [8]. The total phenolic (TPC) and total flavonoid (TFC) content of each extract was determined by photometric methods and their ability to inhibit the digestive enzymes α-amylase and α-glucosidase, DPP IV (an indirect modulator of insulin secretion), and the glycogen metabolism enzyme GPα was investigated in vitro, representing some parts of the human glucose metabolism.

## 2. Materials and Methods

### 2.1. Chemicals and Enzymes

Chemicals were of analytical grade and obtained from Sigma-Aldrich (Taufkirchen, Germany) unless otherwise stated. Sodium dihydrogen phosphate dihydrate was purchased from Riedel de Haen (Berlin, Germany). Magnesium sulfate heptahydrate, disodium hydrogen phosphate monohydrate, and potassium hydrogen phosphate were obtained from Merck (Darmstadt, Germany). NADP disodium salt as well as glycogen from oysters and TRIS HCl were from Carl Roth (Karlsruhe, Germany). DMSO was obtained from J&K Scientific (Marbach/Neckar, Germany). Hydrochloric acid (HCl) and ethanol were purchased from CHEMSOLUTE^®^, Th. Geyer GmbH & Co. KG (Renningen, Germany). Methanol, acetonitrile, and acetone (HiPerSolv CHROMANORM per HPLC) were obtained from VWR (Darmstadt, Germany). Gallic acid and glucose-6-phosphate-dehydrogenase from Saccharomyces cerevisiae (G6PDH, EC 1.1.1.49) were purchased from Alfa Aesar (Haverhill, MA, USA) and formic acid from J.T. Baker (Radnor, Pennsylvania). Ethyl acetate was obtained from Honeywell (Morristown, NJ, USA) and strata C18-E SPE (solid phase extraction) cartridges (55 µm, 70 Å; 20g/60 mL) were purchased from Phenomenex (Torrance, CA, USA). 2-Chloro-4-nitrophenyl-α-D-malto-trioside (CNPG3) and Gly-Pro-7-amido-4-methylcoumarin hydrobromide (H-Gly-Pro-AMC) were obtained from Carbosynth (Berkshire, United Kingdom). 4-Nitrophenyl-β-D-glucopyranoside (pNPG) was purchased from Acros Organics (Fair Lawn, New Jersey). Human dipeptidyl peptidase IV, native enzyme (DPP IV, EC 3.4.14.5) was purchased from Active Bioscience (Hamburg, Germany). The drugs used as positive controls were Januvia 100 mg in which the active compound is Sitagliptin from MSD (Haar, Germany) and Glucobay^®^100 in which Acarbose is the active compound from Bayer Pharmaceuticals (Leverkusen, Germany). α-Amylase from hog pancreas (EC 3.2.1.1), glycogen phosphorylase α from rabbit muscle (GPα, EC 2.4.1.1), α-glucosidase from Saccharomyces cerevisiae (EC 3.2.1.20), and phosphoglucomutase from rabbit muscle (PGM, EC 5.4.2.2) were purchased from Sigma-Aldrich (Taufkirchen, Germany).

### 2.2. Plant Material

Three different batches of Brewer´s spent grain (BSG) were provided by the conventional Orval brewery in Belgium (Florenville, Belgium, BSG 3) and the brewing group of the chair of bioprocess engineering at the Technische Universität Kaiserslautern (Kaiserslautern, Germany; BSG 1,2). The malt used for each brewing process is specified in Table 1. The BSG samples were lyophilized, finely ground with a grain mill, and stored at −20 °C before extraction.

### 2.3. Preparation of Extracts

Three different extraction processes were used including solid-liquid extraction with 60% acetone [24] or ultrasound-assisted alkaline hydrolysis with sodium hydroxide (4 M) [25]. Purification was done by ethyl acetate extraction [26] and solid phase extraction (SPE) with C18e material [27]. In total, seven A (acetone) extracts were prepared by solid-liquid extraction with 60% acetone (acetone/water: 60/40; *v*/*v*), six HE (hydrolysis and ethyl acetate) extracts were prepared by alkaline hydrolysis followed by ethyl acetate extraction, and three HA (hydrolysis and acetone) extracts were prepared by alkaline hydrolysis followed by 60% acetone (acetone/water: 60/40; *v*/*v*) extraction (Table 2). Each extraction process was performed independently and numbering of extracts refers to the extraction process carried out. An overview of the extraction processes is presented in the supplements (Scheme S1). Additionally, some BSG samples were defatted before extraction. This was done by reflux extraction with isopropanol overnight in a ratio of 1 g solid per 11 mL of extraction solvent.

#### 2.3.1. First Extraction Process

The three different BSG samples (BSG 1-3) were extracted twice with 60% acetone (acetone/water: 60/40; *v*/*v*) at 60 °C for 30 min while stirring in a ratio of 1 g solid per 20 mL extraction solvent. The solid residues were separated from the liquid supernatants by filtration, and the residues and filtrates were processed independently thereafter.

After filtration, acetone was removed from the filtrates by rotary evaporation under reduced pressure at 40 °C. The viscous residues were transferred to water (around 50 mL) and methanol was added (around 5 mL) to produce the ‘liquid´ raw extracts A1-A3. These were stored in the dark at −20 °C until purification (see Section 2.3.4).

The solid residues of the initial filtration process were subjected to alkaline hydrolysis. For this purpose, 4 M NaOH was mixed with the samples in a ratio of 1 g solid per 27 mL NaOH and extraction was performed for 90 min in an ultrasonic bath (continuous operation, filled with water until samples were completely covered, 240 watts/period; Bandelin Sonorex RK 106, Bandelin, Berlin, Germany). Afterwards the samples were centrifuged (10 min, 2000× *g*) and the supernatants were adjusted to a pH ≤ 2 with concentrated hydrochloric acid (HCl). The samples were then centrifuged again (10 min, 2000× *g*) and the supernatants were extracted three times with 200 mL of ethyl acetate, which was then removed by rotary evaporation under reduced pressure at 40 °C. The viscous residues were transferred to water (around 50 mL), methanol was added (around 5 mL) to obtain ´liquid´ raw extracts HE1-HE3, and they were stored in the dark at −20 °C before purification by SPE (see Section 2.3.4).

#### 2.3.2. Second Extraction Process

Two different BSG samples (BSG 2 + 3) and one BSG sample previously defatted with isopropanol (BSG 3) were subjected to alkaline hydrolysis with 4M NaOH at a ratio of 1 g solid per 10 mL solvent over 90 min in an ultrasonic bath (continuous operation, filled with water until samples were completely covered, 240 watts/period; Bandelin Sonorex RK 106, Bandelin, Berlin, Germany). The solid residues were separated from the liquid supernatants by filtration, and the filtrates and residues were processed independently thereafter.

The filtrates were adjusted to a pH ≤ 2 with concentrated HCl and then filtered, after which the resulting filtrates were extracted three times with 325 mL of ethyl acetate. The extraction solvent was then removed by rotary evaporation under reduced pressure at 40 °C. The viscous residues were transferred to water (around 50 mL) and methanol was added (5 mL) to obtain the ‘liquid’ raw extracts HE4-HE6. These were stored in the dark at −20 °C until purification by SPE (see Section 2.3.4).

The solid residues of the alkaline hydrolysis were twice subjected to solid-liquid extraction with 60% acetone (acetone/water: 60/40; *v*/*v*) at a ratio of 1 g solid per 10 mL of extraction solvent at 60 °C (first for 30 min, then overnight) by stirring. Afterwards, the combined acetone phases were adjusted to a pH ≤ 2 with concentrated HCl, the samples were filtered, and the extraction solvent was removed by rotary evaporation under reduced pressure at 40 °C. Again, the viscous residues were transferred to water (around 50 mL) and methanol was added (around 5 mL) resulting in ´liquid´ raw extracts HA1-HA3 that were stored in the dark at −20 °C until purification by SPE (see Section 2.3.4).

#### 2.3.3. Third Extraction Process

A third extraction process similar to extraction process 1 was also used. Few modifications were included regarding the grinding grade of the BSG samples as well as the extraction volume used. Two BSG samples (BSG 2 + 3) and two defatted BSG samples (BSG 2 + 3) were milled into a powder and then subjected to solid-liquid extraction with 60% acetone (acetone/water: 60/40; *v*/*v*). A ratio of 1 g solid per 10 mL extraction solvent was used and extraction was performed twice for 30 min each at 60°C under stirring. The samples were then filtered and the extraction solvent of the filtrates was removed by rotary evaporation under reduced pressure at 40 °C. The viscous residues were transferred to water (around 50 mL) and methanol was added (around 5 mL), giving the ‘liquid’ raw extracts A4-A7. These extracts were stored in the dark at −20 °C until purification by SPE (see Section 2.3.4).

#### 2.3.4. Purification by Solid Phase Extraction (SPE)

The ´liquid´ raw extracts obtained after extraction processes 1–3 (A1-A7, HE1-HE6, HA1-HA3) were purified by applying a modified variant of a previously reported solid phase extraction (SPE) method [27] using Strata C18-E g/60 mL cartridges from Phenomenex (Torrance, California) preconditioned with 120 mL of 1% acetic acid in methanol and equilibrated with 120 mL of aqueous 1% acetic acid. The liquid raw extracts were transferred onto the preconditioned cartridges and washed with 180 mL of 1% aqueous acetic acid. Unless the washing solution was clear, the cartridges were then washed with a further 180 mL of 1% aqueous acetic acid. Elution was performed with 60–90 mL of 1% acetic acid in methanol. The amount of elution solvent depended on the extent to which the sample had adsorbed onto the C18e material. Finally, the sample volume was reduced by vacuum centrifugation and the samples were transferred into double distilled water before lyophilization. The extracts obtained after lyophilization (Table 3) were homogenized and stored in the dark at −20 °C until use. Yields are given in Section 3.1.

### 2.4. Total Phenolic Content (TPC)

The total phenolic content (TPC) of the extracts was determined by the spectrophotometric method of Folin-Ciocalteau [28,29] with slight modifications. A calibration curve was generated using reference solutions of gallic acid in dimethyl sulfoxide (DMSO) with concentrations ranging from 20–200 µg/mL gallic acid. In brief, 10 µL of diluted extract (500 µg/mL), gallic acid solution, or DMSO (negative control) and 100 µL of 10% Folin-Ciocalteau reagent were mixed in a 96-well microplate. The samples were incubated for 5 min at room temperature after which 80 µL of sodium carbonate solution (100 g/L) or 80 µL of double distilled water (blank) was added. After 2 h incubation in the dark at room temperature, the absorbance at λ = 750 nm was measured with a microplate reader (Biotek, Bad Friedrichshall, Germany). The TPC was expressed in units of micrograms of gallic acid equivalents per milligram of dry extract (µg GEq/mg extract).

### 2.5. Total Flavonoid Content (TFC)

The total flavonoid content (TFC) of the extracts was determined by a modified aluminum chloride assay [30]. A calibration curve was generated using reference solutions of catechin in DMSO with concentrations ranging from 20–200 µg/mL catechin. In brief, 50 µL of diluted extract (1–5 mg/mL), reference catechin solution, or DMSO (negative control) was added to a well in a 96-well microplate. Aqueous sodium nitrite solution (3%, 20 µL) was then added and the samples were incubated in darkness for 5 min at room temperature. Subsequently, 20 µL of an aqueous aluminum chloride solution (2%) or double distilled water (blank) was added together with 120 µL of double distilled water, and the sample was again incubated for 6 min in darkness. Its absorption at λ = 510 nm was then measured with a microplate reader (Biotek, Bad Friedrichshall, Germany) and the resulting absorptions were used as blank values. Finally, 20 µL of sodium hydroxide solution (1 M) was added and the microplate was incubated on a well plate shaker for 20 min. The absorption at λ = 510 nm was then measured once again and the blank values were subtracted. The TFC was expressed in units of micrograms of catechin equivalent per milligram of dry extract (µg CEq/mg extract).

### 2.6. Inhibition of α-amylase

The α-amylase inhibition assay was based on previously described spectrophotometric methods [31,32]. Samples were measured in triplicate. Acarbose (five concentrations ranging from 0.15–0.55 mg/mL in double distilled water) was used as a positive control, while double distilled water and DMSO were used as negative controls. Extracts were dissolved in DMSO; the highest tested concentration was 10 mg/mL. Briefly, 20 µL of the sample, the positive control, and the negative control were transferred to a 96-well microplate and were each mixed with 70 µL of porcine pancreatic α-amylase (30 U/mL) dissolved in 40 mM phosphate buffered saline (PBS, pH 6.9). Each sample was analyzed alongside a blank (which was mixed with PBS instead of the enzyme solution), allowing each sample’s intrinsic colors to be accounted for during the inhibition calculation. The samples were pre-incubated for 10 min at 37 °C followed by the addition of 100 µL substrate solution consisting of 4 mM 2-chloro-4-nitrophenyl-α-D-malto-trioside (CNPG3) in 40 mM PBS (pH 6.9). The mixtures were then incubated for 8 min at 37 °C and their absorbance was measured at λ = 405 nm using a microplate reader (Biotek, Bad Friedrichshall, Germany). Concentrations and IC_50_-values (half-inhibitory concentrations) were calculated relative to the final concentrations in each well.

### 2.7. Inhibition of α-glucosidase

The α-glucosidase inhibition assay was conducted according to Berger et al. (2020) and You et al. (2011) [31,33]. Samples were assayed in triplicate. Acarbose (five concentrations in double distilled water ranging from 0.4–2 mg/mL) was used as a positive control; double distilled water and DMSO were used as negative controls. Extracts were dissolved in DMSO at a concentration of 10 mg/mL and five different dilutions of these initial solutions (depending on the inhibition strength) were prepared to calculate each extract’s IC_50_ value. Then, 20 µL of each sample, positive and negative controls, were placed in a well of a 96-well microplate and mixed with 70 µL of α-glucosidase from Saccharomyces cerevisiae (1 U/mL) dissolved in 0.1 M PBS (pH 6.8). A blank (in which the enzyme solution was replaced by PBS) was analyzed alongside each sample, allowing the samples’ intrinsic color to be accounted for in the inhibition calculation. After incubation at 25 °C for 10 min, the substrate solution, 4 mM 4-nitrophenyl-β-D-glucopyranoside (pNPG) in 0.1 M PBS at pH 6.8, was added and the samples were incubated again for 5 min at 25 °C. Finally, the absorbance was measured at λ = 405 nm using a microplate reader (Biotek, Bad Friedrichshall, Germany). Concentrations and IC_50_ values were calculated relative to the final concentrations in each well.

### 2.8. Inhibition of Dipeptidyl Peptidase IV (DPP IV)

Inhibition of DPP IV was measured using a modification of the fluorometric method reported by Connolly et al. 2014 [23]. Samples were measured in triplicate. Sitagliptin was used as positive control at five concentrations ranging from 20–60 ng/mL in 20 mM TRIS HCl buffer, pH 8 (TRIS). DMSO and TRIS were used as negative controls. Extracts were dissolved in DMSO at a concentration of 10 mg/mL. Then, 20 µL of the sample, the positive control, and negative control were transferred to a black 96-well microplate for fluorescence measurement with 60 µL TRIS and 100 µL of substrate solution (0.2 mM H-Gly-Pro-AMC in TRIS). DPP IV (20 µL of a 6 mU/mL solution in TRIS) was then mixed into each sample. A blank (in which the enzyme solution was replaced by TRIS) was analyzed alongside each sample, allowing the samples’ intrinsic color to be accounted for in the inhibition calculations. The samples were incubated for 30 min at 37 °C and their fluorescence was read at λ = 360/40 nm (extinction) and λ 460/40 nm (emission) using a microplate reader (Biotek, Bad Friedrichshall, Germany). Concentrations and IC_50_-values were calculated relative to the final concentrations in each well.

### 2.9. Inhibition of Glycogen Phosphorylase α (GPα)

The extracts’ inhibitory potential against GPα was determined by a spectrophotometric method that was adapted for use with 96-well microplates [34]. Each sample was assayed in triplicate. Caffeine solutions (five concentrations in double distilled water ranging from 0.5–2 mg/mL) were used as a positive control; DMSO or double distilled water were used as negative controls. Extracts were dissolved in DMSO at a concentration of 10 mg/mL.

Two buffers were used. Buffer A (3 mM adenosine monophosphate, 40 mM ß-glycerophosphate, 8 mM l-cysteine free base at pH 6.8) was used to prepare GPα stock solutions, while Buffer B (20 mM sodium dihydrogen phosphate dihydrate, 2 mM magnesium sulfate heptahydrate; pH 7.2) was diluted with double distilled water and used as the assay buffer.

Stock solutions of the enzymes were prepared as follows: GPα was dissolved in buffer A to a concentration of 5 U/mL. PGM and G6PDH were dissolved in double distilled water to a concentration of 500 U/mL. All three enzyme stock solutions were stored at −80 °C until use.

The assay medium was prepared by mixing 5 U/mL G6PDH, 12 U/mL PGM, and 3.148 mg/mL NADP in assay buffer. The enzyme mix consisted of 93.75 mU/mL GPα and 100 mM glucose in assay buffer. Glycogen was dissolved in assay buffer at a concentration of 2 mg/mL.

For the assay, 20 µL of the sample (five concentrations depending on inhibition strength), positive control, and negative control were transferred to a 96-well microplate and each mixed with 50 µL of the assay medium. Each sample was then mixed with 80 µL of enzyme mix. Each sample was analyzed alongside a blank (100 mM glucose dissolved in buffer B instead of enzyme mix) to allow the samples’ intrinsic color to be accounted for when calculating the degree of inhibition. The reaction was started by adding 50 µL glycogen (2 mg/mL) and the samples were incubated for 30 min at 37 °C. Finally, their absorbance at λ = 340 nm was measured. Concentrations and IC_50_ values were calculated relative to the final concentrations in each well.

### 2.10. Statistical Analysis

Results are presented as means and SD of three to 83 independent experiments. Statistical analyses were performed with Origin 2019G (OriginLab, Northampton, MA, USA) and Excel Office Professional Plus 2016 (Microsoft, Redmond, DC, USA). Data were checked for normality (Anderson Darling test) and homogeneity of variance (Fisher test). The significance of differences from positive controls (DPP IV and GPα inhibition assays), between extraction groups (TPC assay and GPα inhibition assay), and within extraction groups (TPC and TFC assay, GPα and α-glucosidase inhibition assay) was evaluated using the one-sample *t* test (one-sided). Differences were considered significant at the *p* < 0.05, *p* < 0.01, and *p* < 0.001 levels.

## 3. Results

### 3.1. Characterization of Extracts

The 16 different extracts (A1-A7, HE1-HE6, HA1-HA3) were prepared from three different BSG batches (numbered 1 to 3) from two different breweries. Two of the samples were defatted with isopropanol before extraction. The extraction processes involving SPE purification provided relatively low yields in the range of 0.8–3.5 g/100 g dried BSG (see Table 4). The yields for the different extraction processes were relatively similar ranging from 0.8 to 2.0 g/100 g dw for HE extracts, 0.8 to 2.3 g/100 g dw for A extracts, and 2.5 to 3.5 g/100 g dw for HA extracts. This might be due to the relatively large number of purification steps (particularly the SPE step), which were needed to concentrate phenolic compounds and to eliminate interfering compounds such as sugars that might be released from the lignocellulosic material.

**Figure 1:**
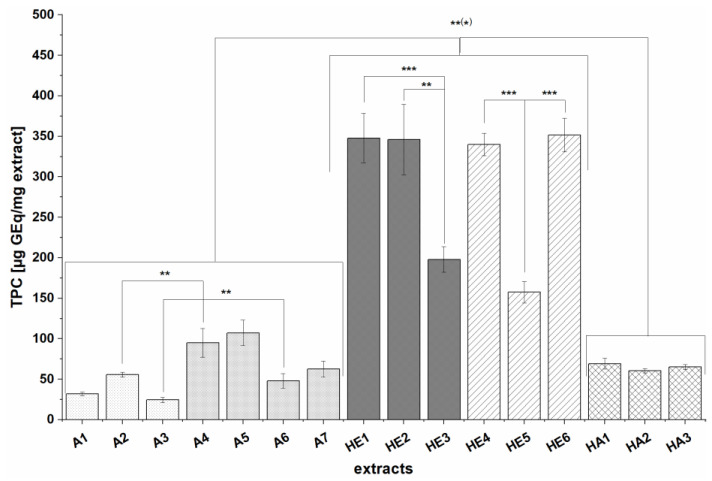
TPC (total phenolic content) of extracts expressed in units of µg GEq/mg extract. Values are expressed as means ± SD of three to five independent experiments each performed in triplicate; significant differences between and within different extract groups were analyzed: ** *p* < 0.01, *** *p* < 0.001.

The TPC values of each extract are shown in Figure 1. All extracts contained detectable amounts of phenolic compounds, ranging from 24.6 ± 3.3 to 351.5 ± 20.7 µg GEq/mg extract. The highest TPCs were detected in extracts prepared by alkaline hydrolysis (HE1-HE6), which contained significantly (*p* < 0.01 and *p* < 0.001) more phenolics (157.5 ± 13 to 351.5 ± 20.7 µg GEq/mg extract) than those prepared by solid-liquid extraction with 60% acetone (A1-A7) (24.6 ± 3.3 to 107.2 ± 15.8 µg GEq/mg extract) and those obtained after hydrolysis followed by solid-liquid extraction with 60% acetone (HA1-HA3) (60.5 ± 2.6 to 69.3 ± 6.7 8 µg GEq/mg extract). Additionally, extracts prepared from previously defatted BSG (A5, A7, HE6) had slightly higher TPCs than those from the corresponding non-defatted BSG samples (A4, A6, HE5); this difference was even significant (*p* < 0.001) for the HE extracts. Extracts prepared from the same BSG batches using different extraction processes also had significantly different TPCs: A2 and A3 had significantly (*p* < 0.01) lower TPCs than A4 and A6. Additionally, HE extracts of the non-defatted BSG 3 sample (HE3, HE5) had significantly lower TPCs (*p* < 0.01 and *p* < 0.001) than all other HE extracts (HE1-HE2, HE4, HE6). In general, extracts of BSG 3 (A3, A6, HE3, HE5, HA2) had lower TPCs than those prepared from other BSG batches independently of the choice of extraction process, while alkaline extracts of BSG 1 (HE1) and BSG 2 (HE2, HE4) had similar TPCs. However, the TPCs of solid-liquid extracts of BSG2 (A2) were almost twice those of BSG1 (A1) leading to the assumption that, besides the extraction process, the BSG batch is also an important factor in terms of TPC.

To compare and discuss our results with literature, the obtained TPC values are related to the yields of the extracts. As our SPE method was only used as a purification step and no single substances were yet identified, no recovery rates were determined. Therefore, the relation to the yields is considered as an estimation giving adjusted TPCs ranging from 0.43 ± 0.08 to 0.86 ± 0.13 mg GEq/g BSG dw for solid-liquid extracts A1-A7, 1.7 ± 0.17 to 2.3 ± 0.11 mg GEq/g BSG for acetone extracts of hydrolysis residues (HA1-HA3), and 2.52 ± 0.21 to 3.96 ± 0.32 mg GEq/g BSG dw for extracts prepared by alkaline hydrolysis (HE1-HE6). The yield-related TPC values maintain nearly the same trend as the extract-related TPCs whereby HA extracts show slightly higher TPC values when related to the yield.

**Figure 2:**
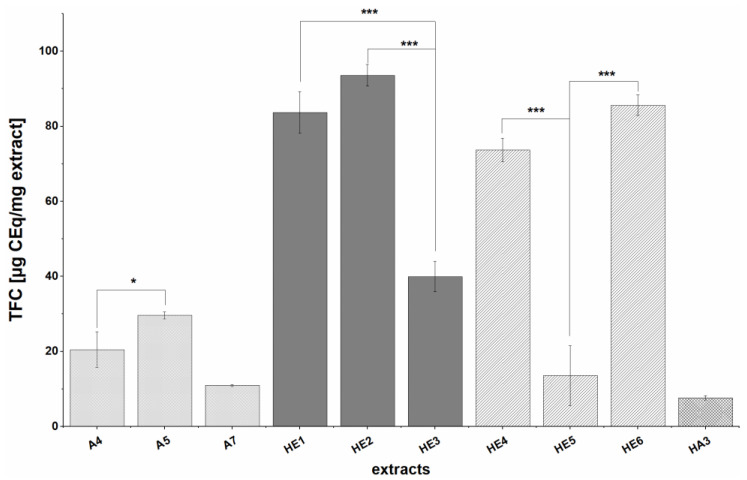
TFC (total flavonoid content) of extracts expressed as µg CEq/mg extract. Values are expressed as means ± SD of three to five independent experiments each performed in triplicate; significant differences within extraction groups are indicated as follows: * *p* < 0.05, *** *p* < 0.001.

The TFC measurements showed that many extracts (A1-A3, A6, HA1-HA2) had no detectable flavonoid content. The other extracts under study (A4-A6, HE1-HE6, HA3) showed TFCs ranging from 7.6 ± 0.7 to 93.6 ± 2.9 µg CE/mg extract (Figure 2). The trends seen for TPCs were also seen for TFCs: extracts prepared by alkaline hydrolysis (HE1-HE6) had higher TFCs (39.9 ± 4 to 93.6 ± 2.9 µg CEq/mg extract) than those prepared by solid-liquid extraction (A4-A5, A7; n.d. to 29.6 ± 0.9 µg CEq/mg extract) or extraction of hydrolysis residues with acetone (HA3; 7.6 ± 0.7 µg CEq/mg extract). In general, the TFCs were around three times lower than the corresponding TPC which could be due to the greater specificity of the flavonoid assay. Additionally, the TFCs of previously defatted samples (A4, HE5) were higher than those of the corresponding non-defatted samples (A5 and HE6; *p* < 0.05 and *p* < 0.001). and as previously observed for the TPC of acetone extracts of defatted BSG, extracts of the defatted BSG batch 2 (A5) tended to have the highest TFC while those from the defatted BSG batch 3 (A7) had the lowest TFC. As was also the case for TPCs, HE extracts from the non-defatted BSG 3 (HE3 and HE5) had significantly lower TFCs (*p* < 0.001) than all other HE extracts (HE1-HE2, HE4, HE6).

As already performed for the TPC values, TFC values were calculated in relation to extracts ’yield to facilitate comparison with previously reported values. The TFCs obtained in this way ranged from 0.16 ± 0.04 to 0.27 ± 0.02 mg CE/g BSG for acetone extracts (A4, A5, A7), 0.27 ± 0.02 mg CE/g BSG for acetone extracts of alkaline hydrolysis residues (HA3), and 0.22 ± 0.13 to 0.94 ± 0.03 mg CEq/g BSG for ethyl acetate extracts of alkaline hydrolysis solutions (HE1-HE5). The same trend as for TPC values was observed; HE extracts had the highest yield-related TFC whereby HA3 extract showed a slightly higher yield-related than extract-related value.

### 3.2. Effects on Enzymes of the Glucose Metabolism

The influence of the different BSG extracts on four glucose metabolism enzymes (α-amylase, α-glucosidase, GPα and DPP IV) was investigated in vitro. The extracts’ inhibitory potential was compared to that of positive controls and expressed as IC_50_ values, i.e., the concentration at which the enzyme’s activity was reduced by 50% if the inhibition was strong enough to calculate this value. Otherwise, the extract’s inhibitory activity was considered slight to moderate.

#### 3.2.1. Inhibition of α-amylase

The effects of the BSG extracts on α-amylase activity were investigated using a spectrophotometric in vitro assay in which the pseudo-tetra-saccharide Acarbose (the active ingredient in the diabetes drug Glucobay^®^100) was used as positive control; its IC_50_ value was determined to be 35.5 ± 4.4 µg/mL. Most extracts did not detectably inhibit this enzyme even at the highest tested concentration of 1.05 mg/mL (this value represents the final concentration of the extract in the incubation solution). However, extracts prepared by solid-liquid extraction with 60% acetone from BSG 1 and BSG 2 (A1-A2, A4-A5) showed slight to moderate inhibition at 1.05 mg/mL, reducing the enzyme’s activity by 23.1 ± 4.2 to 49.7 ± 12.3% (data not shown); this level of inhibition is too low to permit the calculation of an IC_50_ value. Generally, there were some notable discrepancies between the extracts of different BSG batches as well as between the extraction processes. In particular, BSG 3 seems to contain no α-amylase inhibitors, which might be related to the malt used in the brewing process. The active compounds that are present may not be liberated by bases; they appear to be free and easily extractable with 60% acetone since all other extracts (HE and HA) did not show any inhibiting effect.

#### 3.2.2. Inhibition of α-glucosidase

**Figure 3:**
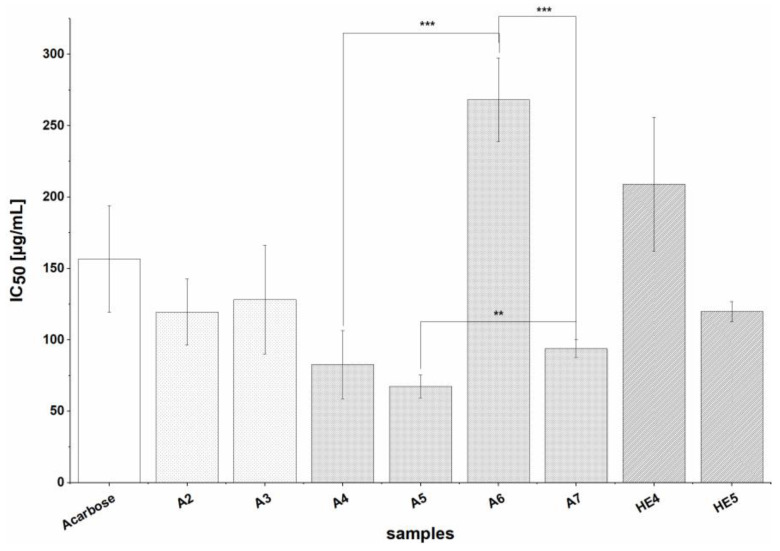
IC_50_ values for the inhibition of α-glucosidase by extracts (A2-A7, HE4-HE5) and the positive control (PC) agent Acarbose. Values are expressed as means ± SD of three to five independent experiments and 83 for PC each performed in triplicate; significant differences within extraction groups are indicated as follows: ** *p* < 0.01, *** *p* < 0.001.

The α-glucosidase-inhibiting potential of the 16 BSG extracts was investigated using the potent inhibitor Acarbose (whose measured IC_50_ value was 156.7 ± 37.3 µg/mL) as a positive control. Extracts A2-A7 and HE4-HE5 exhibited strong inhibition of the enzyme, with IC_50_ values ranging from 67.4 ± 8.1 µg/mL to 268.1 ± 29.4 µg/mL (Figure 3). The other extracts (A1, HE1-HE3, HE6, HA1-HA3) caused only slight (data not shown) or no inhibition and their IC_50_ values could not be calculated. In general, most extracts prepared by solid-liquid extraction with 60% acetone (A) showed inhibitory activity, whereas only two HE extracts (prepared by alkaline treatment followed by ethyl acetate extraction) and no HA extracts (prepared by alkaline treatment followed by extraction with 60% acetone) were strong inhibitors. Additionally, extracts prepared from BSG 2 by solid-liquid extraction with 60% acetone (A4, A5) were significantly stronger inhibitors of α-glucosidase than comparable A extracts from BSG 3 (A6, A7) (*p* < 0.01 and *p* < 0.001). A extracts prepared from defatted material (A5, A7) showed stronger inhibition than those prepared from untreated (non-defatted) BSG samples (A4, A6); this difference was statistically significant when comparing A6 and A7 (*p* < 0.001).

#### 3.2.3. Inhibition of Dipeptidyl Peptidase IV (DPP IV)

**Figure 4:**
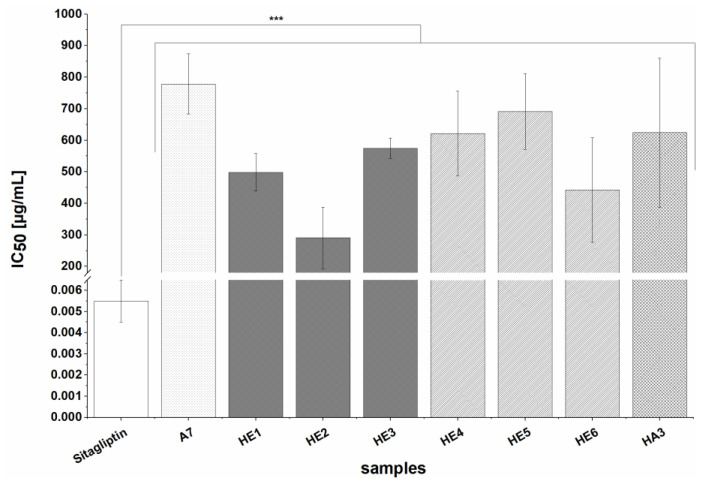
IC_50_ values for the inhibition of DPP IV by extracts (A7, HE1-HE6, HA3) and the positive control (PC) agent Sitagliptin. Values are expressed as means ± SD of three to five independent experiments and 35 independent experiments for the PC, each performed in triplicate; significant differences from the PC are indicated by *** *p* < 0.001.

A fluorometric method using Sitagliptin (in the form of 100 mg Januvia) as a positive control was used to investigate the effects of the BSG extracts (except A2) on DPP IV. The positive control agents resulted in a very low measured IC_50_ value of 5.5 ± 1 ng/mL. All tested extracts inhibited DPP IV at the highest tested concentration of 1 mg/mL. Extracts HE1-HE6 prepared by alkaline hydrolysis and A7 and HA3 (prepared from defatted raw material) strongly inhibited the enzyme, with IC_50_ values ranging from 290.6 ± 97.4 to 778.4 ± 95.5 µg/mL, as shown in Figure 4. All extracts had significantly (*p* < 0.001) higher IC_50_ values than the positive control. Extracts A1 and A3-A6 (prepared by solid-liquid extraction with 60% acetone) and HA1 and HA2 (prepared by acetone extraction of alkaline hydrolysis residues) exhibited slight to moderate inhibition, reducing DPP IV activity by 15.2 ± 4.1 to 49.5 ± 4.4% at a concentration of 1 mg/mL (data not shown). In general, it was mainly HE extracts which showed potent inhibition, whereby the biological relevance has to be evaluated critically due to the potent inhibition of the diabetes drug Sitagliptin.

#### 3.2.4. Inhibition of Glycogen Phosphorylase α (GPα)

**Figure 5:**
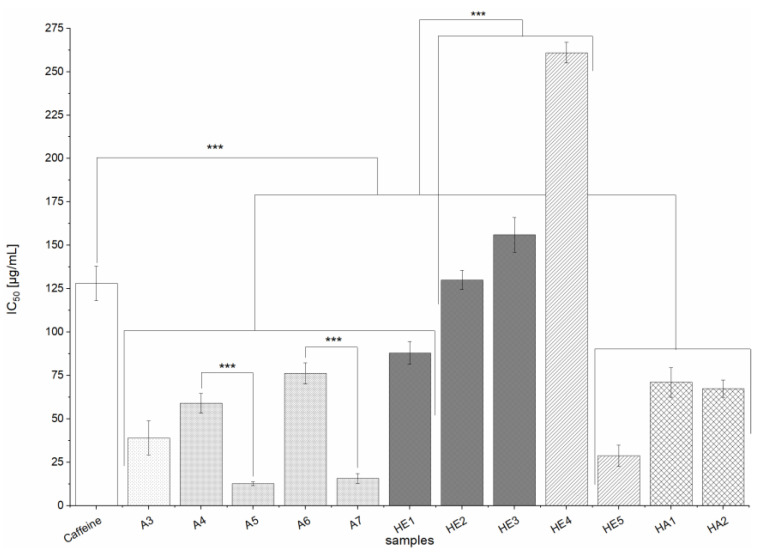
IC_50_ values for the inhibition of GPα by extracts (A3-A7, HE1-HE5, HA1-HA2) and the positive control (PC) agent caffeine. Values are expressed as means ± SD of three independent experiments and 20 for the PC, each performed in triplicate; significant differences between extracts and PC and within and between extraction groups are denoted as follows: *** *p* < 0.001.

The inhibitory effects of the BSG extracts (except A2) on GPα were investigated using a spectrophotometric in vitro assay in which the potent inhibitor caffeine (IC_50_ = 128 ± 10 µg/mL) served as a positive control. All of the tested extracts (A3-A7, HE1-HE5, HA1-HA2) strongly inhibited the enzyme, with IC_50_ values ranging from 12.6 ± 1.1 to 261 ± 6 µg/mL (Figure 5). Extracts A1, HE6 and HA3 were not soluble in the assay medium in the required highest tested concentration of 1 mg/mL, but showed no inhibitory activity at concentrations up to 250 µg/mL. The inhibitory activity of extracts A3-A7, HA1-HA2, and HE1 was significantly stronger than that of the positive control (*p* < 0.001). Furthermore, HE extracts from alkaline hydrolysis (HE2-HE4) were significantly (*p* < 0.001) less inhibiting than all other tested extracts (A3-A7, HA1-HA2, HE1). Additionally, as also seen for the inhibition of α-glucosidase, A extracts made from defatted BSG (A5, A7) were significantly (*p* < 0.001) more potent inhibitors than the corresponding extracts prepared from non-defatted BSG (A4, A6), whereby the difference between the inhibition potential was even higher than as observed for α-glucosidase. In general, extracts prepared by solid-liquid extraction with 60% acetone were more potent regarding the inhibition of GPα than extracts achieved by alkaline hydrolysis (HE extracts). Especially A5 and A7 are of interest, as their effect was about ten times that of the positive control caffeine.

## 4. Discussion

Sustainable and responsible food production has become increasingly important in recent years, leading to an increased emphasis on the productive use of agri-food by-products. Because BSG is rich in fiber and proteins as well as polyphenols such as hydroxycinnamic acids, BSG is both an attractive ingredient for increasing the nutritional value of food [2] and a potential source of bioactive compounds. Hydroxycinnamic acids such as p-coumaric and ferulic acid are already known to inhibit some glucose metabolism enzymes in vitro [14], and the glucose metabolism enzyme DPP IV was inhibited by protein-rich BSG extracts in vitro [23]. Here we studied the in vitro inhibition of four glucose metabolism enzymes (α-glucosidase, α-amylase, DPP IV and GPα) by various BSG extracts. BSG is the main by-product of the brewing process, whose ingredients are water, yeast, malt, and hops. Malt, i.e., barley (*Hordeum vulgare),* is mixed with water at temperatures of around 65 °C in the mashing process, which is followed by the lautering process in which the liquid wort is separated from the solid BSG at a higher temperature (around 75 °C). BSG thus consists mainly of the insoluble components of the barley grains, i.e., the husks, which contain most of the grains’ phenolic compounds [10,35]. During the malting and brewing process, the composition of the barley changes significantly. Although malting facilitates the release and extraction of phenolic compounds and the TPC of BSG is higher than that of the corresponding barley grains, the overall TPC is strongly reduced during the first brewing step [36] In general, the TPC of malt is highly sensitive to the kilning temperature and the presence or absence of hulls [37,38]. Since BSG consists mainly of the barley grains of the malt, the TPC of BSG results to a great extent from the phenolic content in malt used for the brewing.

The extracts used in this study were produced from various BSG batches by three extraction processes including different sequences or combinations of process steps such as solid-liquid extraction with 60% acetone and alkaline hydrolysis with 4 M NaOH. All methods applied have previously been used to extract polyphenols from BSG [24,39]. Alkaline hydrolysis enables the isolation of bound polyphenols, especially hydroxycinnamic acids, because most of the phenolic acids in cereals are ester-linked in cell wall polymers [25]. All three extraction processes were followed by purification steps including liquid-liquid extraction with ethyl acetate for all HE extracts (HE1-HE6). All extracts (A1-A7, HE1-HE6, and HA1-HA3) were subjected to SPE using C18e columns to eliminate interfering compounds and preconcentrate the phenolic compounds [25,27]. The processes we used in our study provided relatively low yields, ranging from 0.8 to 3.5 g/100 g extract, whereby yields were very similar for each extract group Excessive purification steps to eliminate mainly sugars released by alkaline hydrolysis from the lignocellulosic material [40] might explain these results.

The TPCs of the extracts ranged from 24.6 ± 3.3 to 351.5 ± 20.7 µg GEq/mg extract and strongly depended on the extraction method. To facilitate comparison of our results with already reported values in the literature we calculated yield-related values ranging from 0.43 ± 0.08 to 0.86 ± 0.13 mg GEq/g BSG dw for solid-liquid extracts A1-A7, 1.7 ± 0.17 to 2.3 ± 0.11 mg GEq/g BSG for acetone extracts of hydrolysis residues (HA1-HA3) and 2.52 ± 0.21 to 3.96 ± 0.32 mg GEq/g BSG dw for extracts prepared by alkaline hydrolysis (HE1-HE6). The same trend as for extract-related TPC values was observed with HE extracts containing the highest number of polyphenols. This was expected since most of the polyphenols in BSG are esterified and bound to the cell wall, and can thus be released by alkaline hydrolysis or enzymatic pretreatment [8,41,42]. Similar trends, albeit much higher TPC values, have been reported previously—for example, Stefanello et al. found that the TPCs of extracts prepared by alkaline hydrolysis ranged from 12.04 to 17.6 mg GEq/g sample whereas those of solid-liquid extracts obtained using organic solvents were between 1.00 and 3.43 mg GEq/g sample [8]. Similarly, Birsan et al. obtained TPCs of 15.42 to 19.20 mg GEq/g BSG dw for extracts prepared by alkaline hydrolysis and 2.81 to 3.85 mg GEq/g BSG dw sample for solid-liquid extracts prepared with organic solvents. The latter authors also tested liquid-liquid extraction of the hydrolyzed BSG with ethyl acetate; TPCs were much lower (3.08 to 4.71 mg GEq/g BSG dw) than that of hydrolysis-extracts without ethyl acetate extraction but were in the same range than our HE extracts [8,11]. This indicates that reducing ingredients such as sugars are removed by ethyl acetate extraction and that the TPC values are not overestimated in contrast to extracts from alkaline hydrolysis without purification [11,43]. In other studies, TPCs ranging from 0.66 to 9.9 mg GE/g BSG were obtained for extracts prepared by solid-liquid extraction [24,44,45], while analyses of BSG extracts prepared by alkaline hydrolysis yielded TPCs of 10–13 mg GEq/g BSG [46] and 0.014 to 0.732 mg GEq/mL extract [47]. No clean-up by column chromatography or SPE was performed in any of these studies making comparison of TPC values with our results difficult, though at the same time might explain the lower values than those reported in the literature. Regarding the influence of the raw material on the total phenol content, two major points were observed: extracts prepared from defatted BSG (A5, A7, HE6 and HA3) had higher TPCs than the corresponding extracts prepared from non-defatted material (A4, A6, HE5 and HA2), which was already reported for extracts prepared by solid-liquid extraction by Stefanello et al. [8]. Furthermore, differences between the BSG batches were seen; thus BSG 3 extracts generally had lower TPCs than those prepared from other BSG batches independently of the choice of extraction process. As mentioned previously, the TPC of BSG depends strongly on the phenolic content of the malt used in the brewing process which varied for each BSG in our study (see Table 1). Furthermore, the brewing processes differ, which could give rise to differences in the composition of the BSG. For instance, higher temperatures during brewing and the kilning of the malt can lead to the formation of melanoidins via the Maillard reaction [11], resulting in higher TPCs. The differences between the batches could also be partly related to the storage time before lyophilization: BSG is an unstable microbiological material, and due to the structural changes during brewing, it is highly susceptible to microbial attack [48]. The three batches may have been stored for different periods of time before freeze-drying, which would be expected to affect their composition.

The trends observed in the total flavonoid contents (TFC) of the extracts were similar to those observed for the TPC. The measured TFCs were relatively low and depended on the choice of extraction process. Specifically, the TFC values ranged from not detectable (i.e., below the concentration of the most dilute calibration standard, 20 µg/mL) to 93.6 ± 2.9 µg CEq/mg extract Yield related values ranged from 0.16 ± 0.04 to 0.27 ± 0.02 mg CE/g BSG for acetone extracts (A4, A5, A7), 0.27 ± 0.02 mg CE/g BSG for acetone extracts of alkaline hydrolysis residues (HA3), and 0.22 ± 0.13 to 0.94 ± 0.03 mg CEq/g BSG for ethyl acetate extracts of alkaline hydrolysis solutions (HE1-HE5), whereby, as already for the TPC, the same trend as for extract-related TFC was seen. In general, HE extracts (except HE5) had much higher TFCs than A and HA extracts, as was also observed for the TPC. The influence of the raw material was also similar to that in terms of TPC: defatting process (A5, A7, HE6 and HA3) resulted in higher TFCs. In general, the TFCs were around three times lower than the corresponding TPC, which could be due to the greater specificity of the flavonoid assay. Similar trends were observed by Stefanello et al., who used a slightly different method for TFC determination and obtained TFCs of 1.24 ± 0.08 and 1.34 ± 0.03 mg quercetin equivalents (QEq)/g BSG for extracts prepared by solid-liquid extraction with acetone of defatted and non-defatted BSG, respectively, and 2.93 ± 0.22 and 4.54 ± 0.23 mg QEq/g BSG for extracts prepared by alkaline hydrolysis of defatted and non-defatted BSG samples, respectively [8]. In addition, TFCs of 1493.75 ± 91.65 mg QEq/kg BSG have been reported for BSG extracts prepared by solid-liquid extraction with organic solvents [49] and TFCs of up to 44.72 mg QEq/100g fresh weight (fw) were obtained with aqueous solvents [50]. In general, our TFC values are comparable to those reported in the literature for acetone extracts but about 3–4-fold lower than the reported values for those prepared by alkaline hydrolysis. In all of these cases, no clean-up procedures were applied and the method of TFC determination differed from that used in our study which might explain the differences.

After preparing and characterizing the extracts, their biological activity was investigated. Four enzymes catalyzing different stages involved in the glucose metabolism process were studied: the digestive enzymes α-amylase and α-glucosidase, DPP IV, which is an indirect modulator of insulin secretion, and GPα, which is involved in glycogenolysis. All four enzymes are potent targets for the treatment of type two diabetes mellitus [19,51]. The effects of BSG extracts on glucose metabolism have not previously been studied in detail; the inhibitory activity of BSG protein hydrolysates towards DPP IV, α-glucosidase and α-amylase was investigated [23,52], but there are no published studies using extracts such as those examined in this work. However, studies on the inhibition of α-glucosidase and α-amylase by aqueous and organic solvent extracts of barley grains and germinated barley revealed that the extracts inhibited α-amylase more strongly than α-glucosidase and that germinated barley extracts were more potent than those of ungerminated barley [53,54]. Some in vivo studies on the effect of malted barley and barley seed on blood glucose levels have also been reported: Hong and Meang investigated the effect of malted barley in genetically diabetic mice over 12 weeks and observed an insulin-independent 25% reduction in blood glucose levels, together with a reduction in HbA1c levels compared to the control group [55]. Minaiyan et al. reported a sub-acute effect of 75% ethanolic (ethanol/water; 75/25; *v*/*v*) extracts of barley seeds in diabetic rats, leading to a reduction in blood glucose levels after 11 days of consumption [56]. However, it should be noted that the transformation of barley grains into BSG involves several processing steps, which will inevitably lead to significant changes in composition, making these results only minimally comparable to ours. Aside from studies on protein hydrolysates, there is no published data on the effects of BSG extracts on glucose metabolism. We investigated the effects of 16 different BSG extracts on four glucose metabolism enzymes because inhibitory effects have only previously been reported for barley, which is the main ingredient of BSG [57].

None of the studied extracts were found to strongly inhibit α-amylase. Only some extracts prepared by solid-liquid extraction (A1, A2, A4, A5), exhibited slight to moderate inhibition of this enzyme percentage. Thus, neither alkaline hydrolysis nor solid-liquid extraction of alkaline hydrolysis residues enable the extraction of potent α-amylase inhibitors from BSG. However, there were some notable differences between the extracts of different BSG batches. Donkor et al. investigated methanolic extracts of germinated barley with a TPC of around 100 µg ferulic acid equivalents/mL and found that they induced 35% inhibition of porcine pancreatic α-amylase. However, their methodology differed from ours and the concentration of the extract was not reported [53]. In another study using aqueous and ethanolic phenolic barley extracts with TPCs ranging from 0.41 to 0.63 mg GEq/g dw, moderate and strong effects on α-amylase were observed [54]. Although the TPCs of our extracts were higher, we observed no inhibitory effect and inhibitory activity towards α-amylase was uncorrelated with TPC or TFC. This indicates that the phenolic content alone is not predictive of inhibitory activity. However, it may be worthwhile to investigate the inhibitory activity of individual compounds within the extracts. It is possible that the observed inhibition is due to other compounds such as lipids; polar lipids from BSG, beer, and brewing products have been reported to exhibit antithrombotic effects [10], and the oleic and linoleic acid content of mushroom extracts was found to correlate with inhibition of α-glucosidase, and slightly with that of α-amylase [58]. BSG is rich in lipids [9]; accordingly, the lipid content of one of our BSG samples (BSG 3) was around 13.4% (determined by solid-liquid Soxhlet extraction with isopropanol; data not shown). Most lipids in BSG are reported to be triglycerides but there are also around 30% free fatty acids and 9% phospholipids [9]. Acetone is a frequently used solvent for lipid extraction of plant material and BSG [59]. Although pure acetone was used in the work of del Rio et al., aqueous acetone, as used in our extraction processes, may also cause the extraction of some lipids.

Inhibition of α-glucosidase was mainly observed for extracts prepared by solid-liquid extraction with 60% acetone (A2-A7), but two extracts prepared by alkaline hydrolysis (HE4-HE5) also had strong effects on α-glucosidase, with IC_50_ values comparable to that of the positive control agent Acarbose, a well-known inhibitor of α-glucosidase and α-amylase. The other extracts (A1, HE1-HE3, HE6, HA1-HA3) showed moderate inhibitory activity. In general, all of the studied extracts inhibited α-glucosidase in vitro to at least some degree, indicating that BSG is a potent source of inhibitors of this enzyme, whereby mainly A extracts were biological active. Such strong inhibition of α-glucosidase by BSG extracts has not previously been reported; Donkor et al. observed only slight inhibition by methanolic extracts of germinated and ungerminated barley [53], while Ramakrishna et al. reported maximum inhibition values of around 40% for aqueous and ethanolic extracts of barley [54]. The total phenolic contents of our extracts were higher than those used in the two earlier studies, which may explain their stronger effects, but there was no clear correlation between TPC or TFC and strength of inhibition in our studies. It would therefore be useful to investigate the individual components of the extracts to determine their phenolic and flavonoid profiles and identify the compounds active against α-glucosidase.

All of the studied extracts exhibited inhibitory activity towards DPP IV. In particular, all of the alkaline hydrolysis extracts (HE1-HE6) and most of those prepared from defatted samples (A7 and HA3) achieved strong inhibition. Other extracts (A1, A3-A6, HA1-HA2) had only moderate effects. However, when evaluating the relevance of these results, it should be noted that the positive control Sitagliptin (an established anti-diabetic drug) is a 50 to 150 fold stronger DPP IV inhibitor. Most of the extracts studied here were more potent inhibitors than the BSG protein hydrolysates studied by Connolly et al.; while no IC_50_ values were determined by those authors, the highest inhibition observed (using an extract concentration of 1.5 mg/mL) was around 40% [23]. While the effects of our extracts were modest compared to that of the positive control, BSG could be an interesting source of DPP IV inhibitors given that the extracts are complex mixtures and their effects could be due to single compounds present in very small quantities. As also observed in the α-amylase- and α-glucosidase-enzyme assays, there was no clear correlation between TPC and TFC and the strength of inhibition. However, the alkaline hydrolysis extracts (HE extracts) generally had stronger effects on DPP IV than extracts from solid-liquid extraction (A extracts). Given the results of Connolly et al. and the substrate specificity of DPP IV for proteins and peptides, it is possible that amino acids or peptides could be responsible for the inhibitory activity of the BSG extracts. However, the protein content of the alkaline hydrolysis extracts (HA1-HA3 and HE1-HE6) should be relatively low given the nature of the extraction process. Alkaline extraction is a proven method for extracting proteins, which remain in soluble form and can be precipitated and isolated by reducing the pH [60]. In our extraction processes (1 and 2) the pH was adjusted to around 2 but the insolubilized proteins were separated from our supernatant by filtration. Therefore, appreciable levels of proteins and amino acids should only be present in extracts A1-A7, meaning that the DPP IV inhibition caused by the HA and HE extracts is probably due to other compounds.

All of the studied extracts were potent inhibitors of GPα with those prepared with 60% acetone generally revealing stronger effects than those prepared by alkaline hydrolysis (with the exception of HE5). All of these extracts other than HE2-HE4 inhibited the enzyme more strongly than the positive control caffeine. As already seen for α-glucosidase inhibition, extracts prepared from defatted material (A5, A7) were stronger inhibitors than the corresponding non-defatted extracts (A4, A6). GPα inhibition appeared to be uncorrelated with TFC and TPC. However, given the potent inhibitory activity of the extracts (especially A5 and A7, which was around 10 times that of caffeine) BSG seems to be a potent source of GPα inhibitors [61]. As the enzyme has seven binding sites, it has many potential targets for allosteric modulation that can accommodate a wide range of chemical structures. For example, one of the binding sites favors the binding of glucose analogs while another favors heteroaromatic compounds binding [51]. Therefore, many compounds within our extracts could be responsible for the inhibition of GPα.

In general, HE (alkaline hydrolysis followed by ethyl acetate extraction) extracts had significantly higher TPCs and, in most cases, TFCs than A (solid- liquid extraction with acetone) and HA (alkaline hydrolysis followed by acetone extraction) extracts. Nevertheless, HE extracts were only more active than A or HA extracts towards DPP IV. Additionally, the BSG batch had no major effect on the inhibition potential. The acetone extracts were generally more potent inhibitors of α-glucosidase and GPα than the HE or HA extracts, or at least equally strong. For both enzymes, acetone extracts of defatted BSG (A5, A7) were stronger inhibitors than non-defatted acetone extracts (A4, A6). However, the differences between the three BSG batches were minor. It thus seems that the choice of extraction process affects the inhibitory activity of the extracts more than the choice of raw material.

## 5. Conclusions

Multiple batches of BSG were extracted using three different complex extraction processes and their total phenolic as well as total flavonoid contents were determined. The extracts’ effects on the activity of four glucose metabolism enzymes were then investigated. The observed TPCs and TFCs were lower than those reported previously, although it should be noted that SPE cleanup was not applied in previously reported studies on BSG extracts and that both the malt used in the brewing process and the storage conditions of the BSG may affect the extracts’ composition. Several extracts were observed to have strong inhibitory activity, particularly towards GPα and α-glucosidase, but also towards DPP IV. However, the inhibition of DPP IV was considerably weaker than that caused by the positive control, so the biological relevance of the results for this enzyme should be evaluated critically. No appreciable inhibition of α-amylase was observed. In general, the results are not readily compared to literature data because the only relevant previous studies examined BSG protein hydrolysates or barley extracts, and the steps involved in transforming barley grains into BSG (which include malting and the early steps of brewing) will inevitably cause significant changes in the composition of the material [62].

Nevertheless, our results clearly show that BSG is a source of potent inhibitors of various glucose metabolism enzymes, especially GP*α* and *α-*glucosidase, and that further research is warranted to identify the active compounds within these extracts. Additionally, our findings show, that solid-liquid extraction with 60% acetone resulted in more potent extracts regarding GP*α* and *α-*glucosidase inhibition than alkaline hydrolysis. Differences between the BSG batches were of minor importance; the extraction process is crucial for the biological activity determined here. Furthermore, our TPC results confirmed, that bound polyphenols account for the majority of the total phenolics in BSG. However, a high TPC and TFC was no indicator of a more pronounced inhibitory potential. HPLC-DAD and HPLC-MS/MS analysis should be performed to characterize the (phenolic) compounds in the extracts in more detail, and the activity of these compounds should be investigated in enzyme-inhibition assays.

## Figures and Tables

**Table 1:** Brewer´s spent grain (BSG) samples and malt used for brewing.

BSG	Malt Used for Brewing	
1	Wheat malt (54.3%), Pilsen malt (45.7%)	
2	Weyermann^®^ Vienna Malt (100%)	
3	Pilsen malt (90%), caramel malt (9%), peeled, roasted barley (1%)	

**Table 2:** Overview of the extract groups.

Extracts		
60% acetone extraction	hydrolysis and ethyl acetate extraction	60% acetone extraction of hydrolysis residue
purification by solid phase extraction		
A1-A7	HE1-HE6	HA1-HA3

**Table 3:** Overview and nomenclature of produced extracts (purified by SPE (solid phase extraction) and lyophilized).

**Raw Material Used**	**Process 1**		**Process 2**		**Process 3**
	**First Step**: 60% Acetone Extraction	**Second Step**: Alkaline Hydrolysis + Ethyl Acetate Extraction	**First Step**: Alkaline Hydrolysis + Ethyl Acetate Extraction	**Second Step**: 60% Acetone Extraction of Alkaline Residue	60% Acetone Extraction
	Purification by **Solid Phase Extraction**				
BSG 1	A1	HE1	-	-	-
BSG 2	A2	HE2	HE4	HA1	A4
BSG 2 defatted	-	-	-	-	A5
BSG 3	A3	HE3	HE5	HA2	A6
BSG 3 defatted	-	-	HE6	HA3	A7

**Table 4:** Extraction yields of BSG extracts prepared by SPE purification and lyophilization.

Extracts from Solid-Liquid Extraction with 60% Acetone	Yield (g/100 g)	Extract from Alkaline Hydrolysis and Ethyl Acetate Extraction	Yield (g/100 g)	Extracts from Extraction with 60% Acetone of Hydrolysis Residue	Yield (g/100 g)
A1	1.7	HE1	0.8	HA1	2.5
A2	1.2	HE2	1.0	HA2	3.0
A3	2.3	HE3	2.0	HA3	3.5
A4	0.8	HE4	0.9	-	-
A5	0.8	HE5	1.6	-	-
A6	0.9	HE6	1.0	-	-
A7	0.9	-	-	-	-

## Data Availability

The data presented in this study are available on request from the corresponding author.

## Supplementary Materials

The following are available online at https://www.mdpi.com/article/10.3390/nu13082696/s1, Scheme S1: Overview flow chart for extract preparation (A1-A7, HE1-HE6, HA1-HA3).
