# Supplementary figures and images for: Influence of Brewer’s Spent Grain Compounds on Glucose Metabolism Enzymes
Source: Nutrients. 2021 Aug 4;13(8):2696. doi: 10.3390/nu13082696 (PMC8399999; doi:10.3390/nu13082696)

Scheme S1: Overview flow chart for extract preparation (A1-A7, HE1-HE6, HA1-HA3)

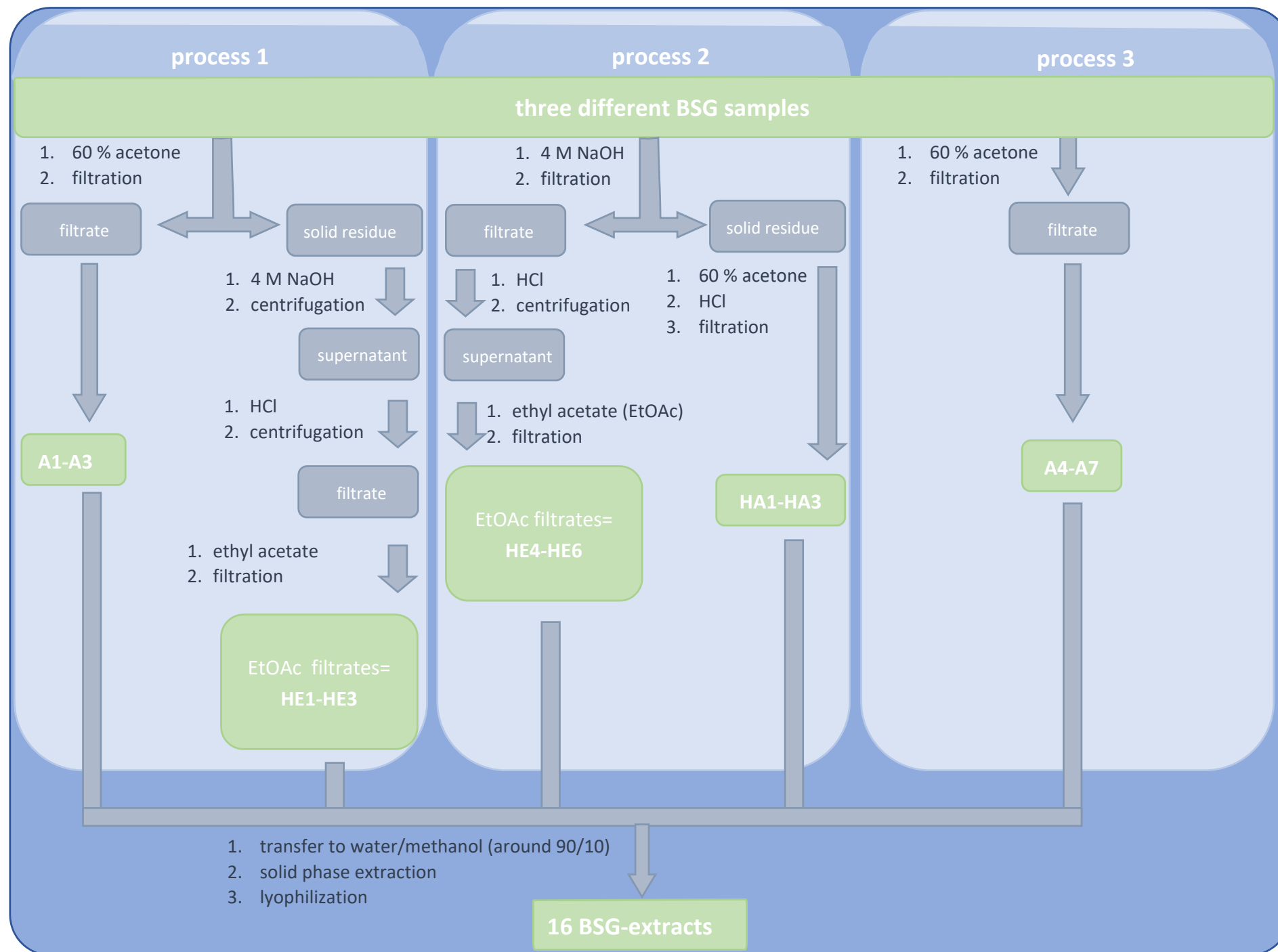

Supplement: Supplementary file 1 [file nutrients-13-02696-s001.zip › nutrients-1316501-supplementary.pdf]
